# Nailfold Videocapillaroscopy as a Candidate Biomarker for Organ Involvement and Prognosis in Patients with Systemic Sclerosis

**DOI:** 10.31138/mjr.30.1.48

**Published:** 2019-03-28

**Authors:** Argyro Repa, Nestor Avgoustidis, Nikos Kougkas, George Bertsias, Michalis Zafiriou, Prodromos Sidiropoulos

**Affiliations:** Department of Rheumatology, Clinical Immunology and Allergy, University of Crete School of Medicine, Heraklion, Greece

**Keywords:** Systemic sclerosis, nailfold videocapillaroscopy, outcome, organ involvement, pulmonary fibrosis, hospitalization

## Abstract

**Background:**

Systemic Sclerosis (SSc) is a rare, multisystemic connective tissue disease associated with significant morbidity. Early recognition of patients at risk for adverse prognosis may help towards optimized monitoring and treatment, thus improving disease outcome.

**Objective:**

To correlate nailfold videocapillaroscopy (NVC) findings (‘early’, ‘active’, ‘late’ scleroderma patterns and non-specific capillary abnormalities) with major organ involvement and prognosis in patients with systemic sclerosis (SSc).

**Methods:**

Patients from the Scleroderma cohort followed at the Rheumatology clinic of the University Hospital of Heraklion will be included. The study will include a prospective and a retrospective part. *Prospective part*: All newly diagnosed patients will undergo NVC at baseline and subsequently every six months. We will review demographics, clinical features and autoantibodies status. Major organ involvement will be monitored (Pulmonary Function Test, DLCO, heart echocardiogram, chest XR, modified Rodnan skin score) at baseline and then every 6–12 months. *Retrospective part*: Existing SSc patients with available NVC data at diagnosis will be included. We will correlate the NVC findings at the time of diagnosis with disease outcomes such as major organ involvement, end stage organ failure, need for hospitalization, and death. We will also correlate longitudinal changes of the NVC patterns with treatment responses and outcomes.

## INTRODUCTION

Systemic sclerosis (SSc) is a rare, multisystemic connective tissue disease associated with significant morbidity. We have previously reported on mortality of SSc in Crete, Greece over a 5-year period (2010–2015).^1^ Specifically, case fatality rate was 9.7% (CI 95% 7.4–11.9) with an average (±standard deviation) age of death at 65.2 (± 17.6) years. Mortality cases were related to SSc in 30.7%. The main cause of death was sepsis (30.8%), followed by pulmonary arterial hypertension (PAH) and cardiac arrest (15.4% each). Early identification of subgroups of SSc individuals at increased risk for severe organ involvement and poor prognosis may be helpful to optimize monitoring and treatment in order to improve SS outcome.

Nailfold videocapillaroscopy (NVC) represents a safe, non-invasive method to detect and analyze morphological microvascular abnormalities in SSc patients. Cutolo et al. classified the findings of capillaroscopy in SSc patients into early, active and late patterns. The *early SSc pattern* is characterized by the presence of a small number of giant capillaries and capillary microhemorrhages, no evident loss of capillaries and relatively well-preserved capillary distribution. The *active SSc pattern* is characterized by larger number of giant capillaries and capillary microhemorrhages, moderate capillary loss, and mildly disorganized capillary architecture. Finally, *the late SSc pattern* is characterized by the near-absence of giant capillaries and microhemorrhages but involves severe loss of capillaries and the development of extensive avascular areas, ramified and bushy capillaries (indicative of neo-angiogenesis), and severe disorganization of the normal capillary array.^2^ Of note, data from the EULAR scleroderma trials and research (EUSTAR) database have shown that in 13.7% of SSc patients, the capillaroscopy pattern was not classified as “scleroderma pattern”. Normal pattern was reported in 27.3% and non-specific capillary abnormalities were reported in 55.5% of those patients.^3^

NVC is accepted as a diagnostic tool and addition of nailfold capillary abnormalities to the ACR SSc classification criteria was shown to improve their sensitivity. As a result, abnormal nailfold capillary pattern consistent with SSc has been included as one of the ACR/EULAR 2013 classification criteria.^4^

Subsequent studies have identified associations between NVC changes and a number of clinical features. Smith et al. reported that the odds ratio (OR) for future severe peripheral disease for early, active and late pattern versus normal pattern was 2.49, 6.18 and 15.35, respectively. The OR for future severe lung involvement was 2.54 for early, 6.43 for active and 16.30 for late pattern, suggesting that capillaroscopy could be used as a biomarker.^5^ Paxton et al. performed a systematic literature review to evaluate the prognostic value of NVC in predicting disease outcomes in SSc patients and demonstrated an association between NVC abnormalities, especially capillary loss, and disease severity (particular vascular manifestations such as DU, calcinosis and PAH).^6^

Avouac et al. also evaluated changes of capillaroscopy findings over three years in order to detect associations with overall disease progression and organ involvement. They reported significant NVC changes in 51% of SSc patients. Higher number of giant capillaries was negative prognostic factor for new digital ulcers (DU) (HR: 0.53), while the loss of capillaries over time was associated with overall disease progression (HR: 4.35), occurrence of new DU (HR: 5.33), lung vascular progression (HR: 18.53), progression of skin fibrosis (HR: 4.22,) and worsening of the Medsger severity score (HR: 5.26). They concluded that sequential capillaroscopy could be used to monitor SSc.^7^

Finally, Soulaidopoulos et al. reviewed the literature regarding the relationship between NVC and visceral organ involvement in SSc. Although there is circumstantial data linking NVC patterns with severe organ involvement, they concluded that the studies are small, cross-sectional cohorts; therefore, better-designed studies are required in order to confirm the relationship between the evolution of microangiopathy and organ specific complications in SSc.^8^

To this end, our aim is to study the associations between NVC findings (‘early’, ‘active’, ‘late’ scleroderma pattern and non-specific capillary abnormalities) with severe clinical involvement and prognosis in a SSc population.

## PATIENTS AND METHODS

Prospective and retrospective study of patients from the Scleroderma cohort followed at the Rheumatology clinic of the University Hospital of Heraklion.

*Prospective study*: We will analyze demographics, clinical features, autoantibodies status and treatment from all patients with newly diagnosed SSc (inception cohort). SSc *sine scleroderma* patients will be analyzed as limited SSc (lSSc) patients. The modified Rodnan skin score (mRSS) will be used to assess skin involvement. Major organ involvement will be monitored every 6–12 months with ECG, Pulmonary function tests (PFTs) including DLCO, chest X-ray and heart ultrasound.

NVC will be performed in 8 fingers using videocapillaroscopy with magnification 200× and the result will be classified as SSc pattern or non-specific capillary abnormalities. SSc pattern will be classified according to the classification proposed by Cutolo. Non-specific capillary abnormalities are characterized by the absence of giant capillaries but the presence of capillary microhemorrhages and/or morphological disorders of the capillaries. NVC will be performed every 6 months and changes in the pattern will be recorded. We will correlate the findings from the NVC with the disease clinical features as well with the treatment, organ involvement, the need for hospitalization and mortality.

The data will be recorded at the existing registry of SSc of the University Hospital of Crete. The prospective part of the study will last for 3 years.

*Retrospective study*: Patients from the Scleroderma cohort who had capillaroscopy at the time of diagnosis will be included. Our registry includes 92 patients with complete laboratory and clinical data. The findings from the NVC will be correlated with demographics, organ involvement and response to treatment.

## IMPORTANCE OF THE STUDY

SSc is a rare disease causing significant morbidity and mortality. Videocapillaroscopy is an easy method to assess the patients. In the present study, we will assess the value of videocapillaroscopic images at diagnosis as a prognostic biomarker for future severe organ involvement in SSc patients. Availability of reliable prognostic biomarkers or clinical indices could lead to tailor early aggressive treatments for those patients with poor prognosis in order to improve SS outcome.

## STUDY APPROVAL

The study has been approved by the Ethics Committee of the University Hospital of Heraklion.
